# Scalable integration of nano-, and microfluidics with hybrid two-photon lithography

**DOI:** 10.1038/s41378-019-0080-3

**Published:** 2019-09-09

**Authors:** Oliver Vanderpoorten, Quentin Peter, Pavan K. Challa, Ulrich F. Keyser, Jeremy Baumberg, Clemens F. Kaminski, Tuomas P. J. Knowles

**Affiliations:** 1 Department of Chemical Engineering and Biotechnology, University of Cambridge, Philippa Fawcett Drive, Cambridge, CB3 0AS UK; 2 Department of Chemistry, University of Cambridge, Lensfield Road, Cambridge, CB2 1EW UK; 3 Cavendish Laboratory, Department of Physics, University of Cambridge, J. J. Thomson Avenue, Cambridge, CB30HE UK

**Keywords:** Nanofluidics, Nanofabrication and nanopatterning

## Abstract

Nanofluidic devices have great potential for applications in areas ranging from renewable energy to human health. A crucial requirement for the successful operation of nanofluidic devices is the ability to interface them in a scalable manner with the outside world. Here, we demonstrate a hybrid two photon nanolithography approach interfaced with conventional mask whole-wafer UV-photolithography to generate master wafers for the fabrication of integrated micro and nanofluidic devices. Using this approach we demonstrate the fabrication of molds from SU-8 photoresist with nanofluidic features down to 230 nm lateral width and channel heights from micron to sub-100 nm. Scanning electron microscopy and atomic force microscopy were used to characterize the printing capabilities of the system and show the integration of nanofluidic channels into an existing microfluidic chip design. The functionality of the devices was demonstrated through super-resolution microscopy, allowing the observation of features below the diffraction limit of light produced using our approach. Single molecule localization of diffusing dye molecules verified the successful imprint of nanochannels and the spatial confinement of molecules to 200 nm across the nanochannel molded from the master wafer. This approach integrates readily with current microfluidic fabrication methods and allows the combination of microfluidic devices with locally two-photon-written nano-sized functionalities, enabling rapid nanofluidic device fabrication and enhancement of existing microfluidic device architectures with nanofluidic features.

## Nanoengineering: Flexible production of micro- and nanofluidic devices

A laser-based manufacturing process can produce combined nanofluidic and microfluidic devices in a rapid and scalable manner. Nanofluidic devices allow for the observation of biological processes at the single-molecule level, but current fabrication methods are slow and costly. In this article, Tuomas Knowles and his team from the UK’s University of Cambridge used conventional light-blocking masks and laser technology to craft a light-sensitive material into devices with micron to nano-sized channels. The researchers used microscopy and single-molecule tracking of dyes to verify the functionality of the final imprinted device, which also demonstrates the integration of nanofluidic channels into existing microfluidics designs. The team says that further research may optimize their new platform, which they hope will offer a reliable and flexible pathway for nanofluidic device fabrication.

## Introduction

Micro and nanofabrication give new possibilities to analyze molecular processes with high precision. Microfluidics^1^ has for instance become a powerful tool to study biological systems at single cell resolution^2–4^ and has allowed the observation of single molecules^5–7^ and of single nucleation events in protein aggregation^8^ in a controllable environment. Microdroplet generation^9^, microfluidic diffusional sizing^10–12^, and electrophoresis on chip^13–15^ are now established techniques, but the nanofluidic regime has the potential to open up a new set of possibilities applications^16–18^. Many specific physical features motivate the interest in nanofluidic device fabrication, including the existence of selective transport mechanisms occurring when channel widths reach diameters close to the Debye length^19,20^. Interactions between protein charges and the Debye layer can be used to confine, separate, and concentrate proteins^21^ as well as sorting exosomes and colloids according to their size down to 20 nm^22^. Moreover, nanofluidics has the potential to provide increased efficiencies in blue energy, where osmotic power is harvested from Gibbs free energy of a salt gradient between two solutions connected by nanopores, nanochannels, or membranes^23^. Performance of reverse electrodialysis depends on the scale and geometry of the nanoscale confinement used, as has been studied^24,25^ in cylindrical and conical shaped nanochannels down to tens of nanometers.

Currently, to produce lab-on-chip devices in the nanofluidic regime, electron beam lithography (EBL) and focused ion beam etching are used in clean room facilities to prototype nanochannels or nanopores in the sub-100 nm range in silicon/silicon nitride^26^. EBL can achieve channel sizes smaller than 10 nm^27^, but cannot pattern as fast as mask-based lithography approaches^28^. These approaches work well, but can be challenging to integrate with microfluidics and are costly and can require long writing times. Here, we demonstrate an approach to produce nanofluidic chips at wafer-scale in a nonclean room environment using a hybrid lithography approach bringing together direct two photon writing for defining nanoscale structures with conventional whole-wafer mask ultraviolet (UV) lithography.

The adoption of microfluidic technologies has been substantially accelerated by soft-lithography approaches^29^ and currently common practice for lab-on-a-chip device fabrication on a laboratory scale is UV mask photolithography followed by soft lithography. There are two main photolithographic strategies to produce master molds for soft lithography—large area mask patterning and direct laser-writing (DLW)^30,31^. In general, both techniques work with UV-curable photoresists such as SU-8 spincoated onto a silicon wafer, with specified thicknesses of tens of microns. Uncross-linked SU-8 is soluble in the developing agent PGMEA but becomes cross-linked and insoluble when exposed to UV radiation and post-exposure heat treatment. UV attenuating masks with transparent sections in areas to be solidified are brought between the light source and the wafer to project microfluidic chip designs onto the photoresist coated wafer. Unilluminated areas are afterwards dissolved during the development process and only the UV-exposed areas remain. The wafer surface then is used as a mold for soft lithography using PDMS casting. DLW has the advantage that there is no need for masks. In this approach a laser is scanned over the wafer and is modulated accordingly to write the intended pattern. Both of these technologies are restricted by the diffraction limit and their patterning capabilities are conventionally limited by the thickness of the photoresist between 5 and 120 µm for common microfluidic fabrication because exposure triggers polymerization throughout the thickness of the photoresist. Two-photon lithography overcomes these limitations by using high power femtosecond pulse laser sources operating at twice the absorption wavelength used for the conventional DLW process. Two such high wavelength low-energy infrared radiation (IR) photons can interact with the photoinitiator molecule if they arrive within the very short lifetime of the virtual state created by the interaction of the first photon with the absorbing molecule in the photoresist^32^. This effect is thus strongly dependent on the power of the incident radiation^33^. In two-photon lithography, polymerization only takes place when the square of the incident power reaches a threshold, unlike for conventional single photon lithography where polymerization is controlled by the intensity itself. Since the volume in which the square of the intensity is above a critical threshold can be smaller than the volume in a diffraction limited focused laser, sub-diffraction limited features can be generated. Considering an additional nonlinear photo response of the photoresist materials itself and adjusting the laser intensity close to the energy threshold for polymerization, SU-8 nanorods of about 30 nm have previously been presented^34^. The fact, that two incoming photons are unlikely to interact with a photoinitiator molecule at the same time before reaching the focal spot opens up arbitrary sectioning capabilities within the spincoated photoresist layer thickness. The application range of two-photon lithography ranges from the fabrication of photonic crystals^35^, cell scaffolds^36^ to metamaterials^37^, biomimicry^38^, and additive manufactured microfluidics^39^. Two-photon lithography has been used^40^ to incorporate optical components (e.g., a total internal reflecting mirror) with a microfluidic channel using soft lithography and to demonstrate the integration of a three-dimensional microfluidic mixer into photolithographically fabricated areas on a glass coverslip^41^. In this paper, we enhance the strategy of combining conventional UV lithography with two-photon direct laser writing to approach the nanofluidic regime on a silicon surface. Silicon wafers are more common for microfluidic master fabrication than glass coverslips, due to their mechanical strength and surface quality. In the following, we introduce and experimentally demonstrate the successful master fabrication of nanofluidic chip devices on a silicon wafer using standard SU-8 with a channel size of 420 nm and show that arbitrary height channel mold fabrication down to 50 nm is possible. A custom-built two-photon setup was characterized with a calibration assay to determine the achievable minimal feature size of the system. Three system parameters were systematically evaluated to define the achievable resolution of the writing process: laser power, scanning speed, and focal spot offset from the wafer surface. To find the optimal values of these, test gratings were written in SU-8 at different conditions. Scanning electron microscopy (SEM) and atomic force microscopy (AFM) for the characterization of the polymerized features and the evaluated parameters, were used to incorporate nanofluidic channels into a microfluidic master. To show the successful imprinting of nanofluidic PDMS devices, their fluidic connectivity was demonstrated by flowing Rhodamine 6G dye through the channels and imaging their lateral width on chip, using super-resolution microscopy. The procedure presented here, fills the gap of affordable nanofabrication in biological laboratories in combination with conventional UV lithography—overcoming low patterning speeds of DLW-technology but achieving subdiffraction features in areas of interest.

## Results

### Combining UV lithography with two-photon DLW for wafer-scale nanofluidic chip fabrication

To explore the integration of nanofluidics with microfluidics we focus on a prototypical nano/micro device, consisting of two microfluidic channels that are connected via nanochannel junctions. These can be used for instance to isolate single molecules from a solution and study their diffusion properties. On one side a sample solution is pumped through, while the other compartment of the device will be exposed to particles or proteins that fit through the nanosized restriction connecting them.

**Fig. 1:**
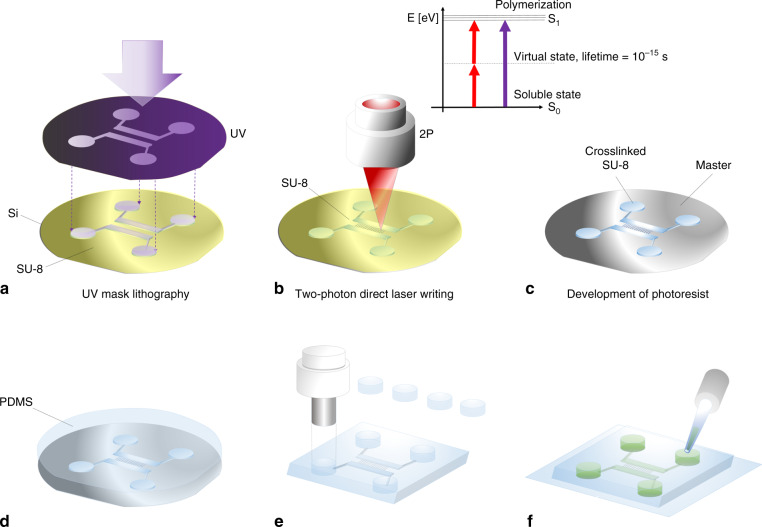
Process outline for nanofluidic device fabrication via combination of two-photon lithography and mask UV lithography. UV lithography; (**a**) mask-based UV lithography is used to project an arbitrary microfluidic chip design onto a SU-8 coated silicon wafer; **b** nanofluidic channels are added via two-photon lithography in areas of interest. The jablonsky diagram illustrates that two spatially correlated IR-photons can interact with the photoinitiator like one single UV photon with half the wavelength if they are absorbed by the molecule within the lifetime of a virtual state excited by a single-IR photon; **c** the wafer is developed and a microfluidic master wafer with integrated nanofluidics obtained; **d** the wafer is covered with PDMS for soft lithography; **e** after curing of the PDMS, devices are peeled off the surface and inlets added; **f** the final PDMS-chip is plasma bonded onto a glass coverslip and filled with, e.g., fluorescent dye

**Fig. 2:**
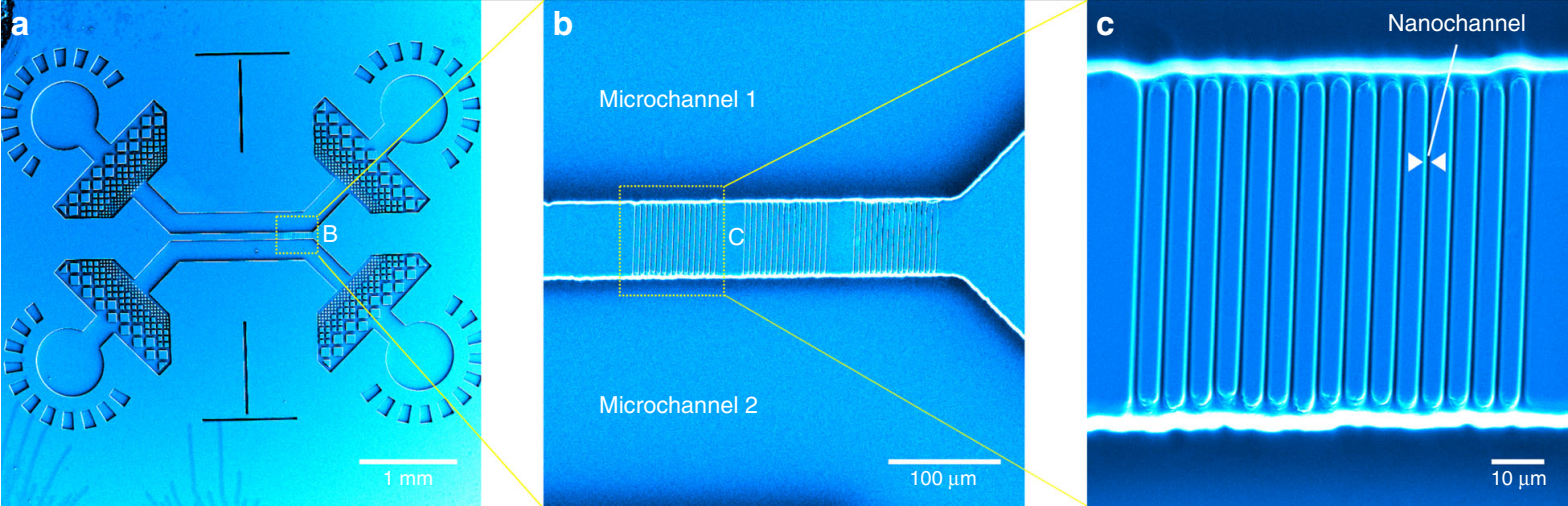
Correlated SEM and AFM analysis of 2-photon nano structures. **a** SEM micrograph of nanofluidic PDMS imprint fabricated by the combination of UV mask lithography and 2-photon writing; **b** three nanofluidic areas with nanochannels of 75 micron length joining the two microchannels; **c** higher magnification shows 420 nm wide nanochannels imprinted in PDMS

Conventional methods to produce such nanofluidic devices are based on spincoating of sub-100 nm thin photoresist films and exposure through UV attenuating masks or require electron beam lithography to push the lateral width down to the nanoscale^42^. Practical limitations of these methods are long writing times and variations in the photoresist thickness that render the integration of nanofluidics difficult. In our procedure presented here (see Fig. 1a–f), we can use a single spin-coating process of a thick (e.g., 25 µm) SU-8 layer to fabricate nanofluidic master molds on a silicon wafer (Supplementary Figs. 1 and 2). The wafer is prebaked to remove solvent and UV-exposed through a film mask to pattern microfluidic areas on a waferscale. The wafer was then postbaked to polymerize the irradiated areas. The baking process induces a refractive index difference between exposed and unexposed areas. These refractive index edges were then used in the two-photon setup to find the regions of interest on the microfluidic master wafer, where the nanojunctions should be written. Since two-photon polymerization is induced only in the focal spot, we have arbitrary control over the height of the printed channels—we overcome the drawbacks of conventional necessary multiple spincoating steps and fabricate devices within one development process. In a second step the master wafer is developed in PGMEA, cleaned with isopropanol and dried using pressurized nitrogen. Through soft lithography, we imprinted the pattern of the functionalized nanofiltration chips in PDMS. The final PDMS imprint with additional microfluidic prefiltration section can be seen in Fig. 2a. SEM images reveal the successful connection of the two microchannels with nanochannels of 420 nm width and 75 microns in length (see Fig. 2b, c). Due to double exposure by UV lithography and two-photon writing the nanochannels show broader joints at the intersection with the microfluidic area.

### Calibration assay for micro-, to nano-scale 2-photon writing

**Fig. 3:**
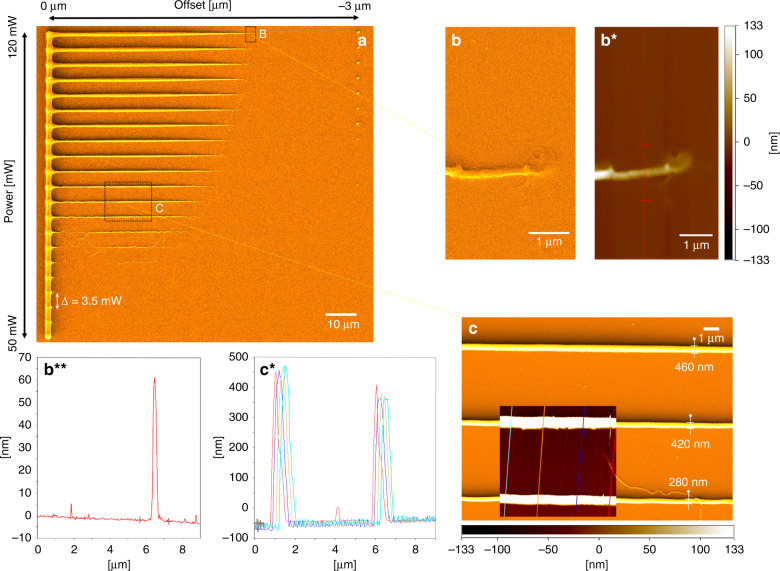
Correlated SEM and AFM analysis of 2-photon nano structures. **a** SEM image of power offset parameter test pattern at a writing speed of 400 µm/s. From bottom to top the power was varied from 50 to 120 mW. Horizontally, from the left to right the 2P-voxel was deepened into the wafer from zero offset—relating to the focal spot being on the wafer surface—to −3 µm, which relates to the focal spot being 3 microns inside the silicon from the wafer surface. One can see that at constant power just by varying the height, the lateral line width can be pushed to the nanoscale; **b** SEM-image of the position where the polymerization inducing voxel is disappearing into the wafer; (B*) Correlated AFM image of position B; (B**) AFM line plot of height profile measurement along red line as indicated in (B*) verifying a size down to 52 nm; **c** detailed SEM imaging verifies soft lithography compatible structures down to 280 nm in width and illustrate how channel size can be controlled at constant offset by variation of power; (C*) AFM height profiles along colored lines as indicated in (**c**)

Since two-photon polymerization is a dose dependent process, the polymerized voxel scales with the intensity and the scanning speed of the laser beam in the photoresist. In addition, since 2PL is used here for 2.5 dimensional fabrication, the voxel truncation by the wafer surface also plays an important role on the lateral size of the written lines. A systematic approach was employed to evaluate the key factors in the system and define suitable operation parameters for micro-, to nano-sized two-photon writing on the wafer surface. Faster scanning speed as well as lower laser intensity were observed to result in a smaller voxel size. Voxel truncation influences both and is furthermore important for the height of the channel molds and proper attachment of the polymerized resin. We evaluated powers ranging from 50 to 120 mW measured after the acousto-optic modulator and ascending of the laser focus into the wafer from 0 to 3 µm at a constant writing speed of 400 µm/s. An SEM image of the calibration print can be seen in Fig. 3a. To improve SEM image quality and contrast, the sample was coated with 10 nm platinum. Along the vertical axis the laser power for each line was varied, where the highest intensity was used at the top line. As expected, with increasing laser power the voxel size increases from bottom to top. At the lower end the effective laser dose did not reach the polymerization energy threshold, which results in no polymerization to occur. From left to right, a decrease in lateral width can be observed due to the laser focus being lowered 3 µm into the wafer over a distance of 100 µm. Using this calibration map, suitable parameters can be read out and the spot size adjusted according to the filling factor of the pattern—or in this case—the channel dimensions to be written. We found soft lithography compatible structures down to a size of 280 nm lateral width as shown by SEM imaging in Fig. 3b and even 230 nm if the optical design is adopted (Supplementary Fig. 3). AFM measurements of the region show heights down to 360 nm and are illustrated in Fig. 3c along with height profile measurements in Fig. 3c*. Two-photon written nanochannels are by nature of hyperbolic shape^32^, assuming a Gaussian intensity distribution in the focal spot, which limits this technology for the fabrication of rectangular channels. In comparison with other nanolithography techniques the achievable lateral widths are relatively large but AFM measurements at positions where the voxel is about to disappear into the sample, verified a height of down to 52 nm (see Fig. 3b*) and 35 nm height (Supplementary Fig. 4). Precise height control of the sample is required to approach fabrication in this regime due to nonlinear behavior of the channel height when truncating the voxel on the surface (Supplementary Fig. 5). We found that high spatial frequency components of the focused laser beam interfere above the wafer surface and result in multiple polymerization locations which can lead to detachment of the structures during the development process. Reducing the beam diameter before the objective resulted in an improvement of fabrication quality—showing similar lateral resolution and smooth lines when ascending the voxel into the wafer (Supplementary Fig. 6).

### TIRF-super-resolution imaging of Rhodamine 6G molecules in nanochannels

**Fig. 4:**
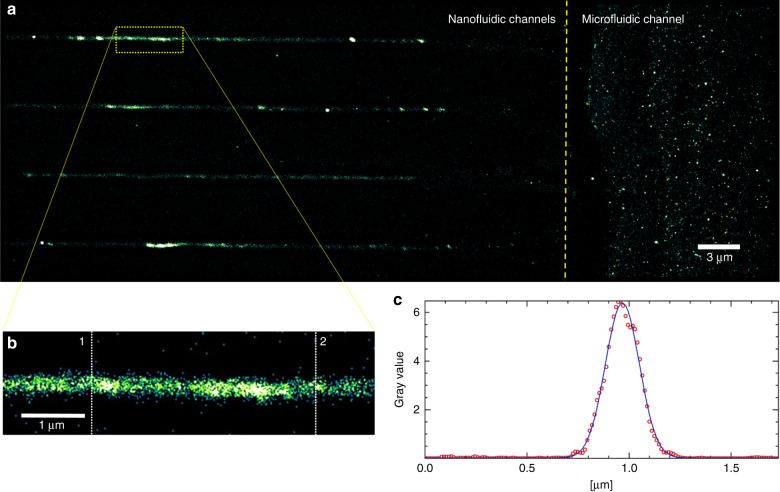
Super-resolution imaging of nanofluidic devices. **a** Super-resolved dSTORM image of a nanofluidic PDMS chip with Rhodamine 6G diffusing through 2-photon written nanochannels. **b** Zoomed in region, verifying the successful imprinting of fluidically connected nanochannels in PDMS; the channel size was measured by averaging the vertical line profiles reaching from position 1 to position 2; **c** plot of averaged line profiles as indicated in (**b**)—verifying the intended channel width of 420 nm on chip and a FWHM of 200 nm. Reconstruction was computed with the ThunderSTORM software plugin for ImageJ.^45^

In order to test the fluidic connectivity of the nanofluidic devices, we flushed from the microfluidic inlet, (A) in Fig. 4, an aqueous solution of a dye. Since the channels’ dimensions are of the order or smaller than the wavelength of light, we used super resolution microscopy to image the nanofluidic channels. To this effect, total internal reflection (TIRF) illumination was used to restrict the excitation area of a fluorescence microscope to an area of approximately 100 nm above the coverslip. Since out of focus fluorophores are not excited, this technique has much higher signal to noise ratio and is perfectly suitable for nanochannel measurements due to spatial confinement of molecules close to the coverslip and allows the observation of single fluorescent molecules diffusing into the nanochannels from the reservoirs (Supplementary video 1). To verify the correct bonding and size consistency of the channels on chip we filled the reservoirs with Rhodamine 6G solution at femto molar concentration dissolved with 200 mM MEA in phosphate-buffered saline. The solution was adjusted with KOH to a pH of 10 in order to induce blinking of the fluorescent dye molecules and enable breaking the resolution limit of conventional fluorescence microscopy by using the STORM principle^43^. By taking thousands of images, localizing the fluorescent emitters in each image and overlaying the data, a super resolved image can be reconstructed. A description of the setup that was used for imaging can be found in Rowlands et al.^44^. Single molecule localization is a useful method for the characterization of nanofluidic devices after the plasma bonding step to verify fluidic connectivity and offers an alternative to clean room equipment such as electron microscopy or AFM. From the super-resolved fluorescence microscopy image the effective channel size was measured to be 200 nm (full width at half maximum) (see Fig. 4a, b), and is consistent with the expected channel size read out from the calibration data acquired by SEM and demonstrates the reliability of this technique. Small distortions induced by sample handling during the bonding process can be visualized on chip and imply the importance of careful handling of the PDMS during the bonding process. The detected molecules show a Gaussian distribution along the channel width, which could be due to the increased channel height toward the nanochannel center. This caused an increase in the amount of detected molecules in the center in comparison to positions close to the walls.

## Discussion

In this paper we show that two-photon lithography can be used to fabricate nanofluidic channels of arbitrary heights from micron to sub-100 nm using materials conventionally employed for microfluidic fabrication and to integrate these structures with microfluidics. Two-photon lithography provides a reliable technique that decouples the influence of varying photoresist thicknesses from nanofluidic fabrication. The method reaches the upper boundary of the ultrananoscale with 35–50 nm channel heights, where new charge induced transport mechanisms start to appear, but remains in lateral resolution one order of magnitude larger than EBL and RIE. Also the round shape of the polymerization voxel inhibits the fabrication of rectangular shaped channels which is achievable using RIE. Another technique, e.g., crack induced nanochannels—provides a simple and nonclean room technique with channel sizes in the sub-100 nm regime. Approaches such as generating mechanically induced cracks in chips have a high fabrication speed and can rapidly generate nanochannels up to mm length. However, this method cannot be easily interfaced with traditional PDMS microfluidics. The combination of EBL and UV lithography is possible, but it is challenging without extending write times and costs, whereas 2PL provides high flexibility for the effective integration of arbitrary two-dimensional nanofluidic functionalities into microfluidic masters. Although two-photon lithography cannot achieve as small features as electron-beam lithography, its fabrication range is useful for a variety of biophysical and blue energy applications, e.g., charge measurements of proteins in solution using nanotraps with dimensions of 600 nm width and 160 nm height^46^. Nano-electroporation uses 90 nm wide channels for precise dose control for the injection of nanoparticles, plasmids and siRNA on a single cell basis and demonstrate the improvement of the process when channels in the submicron regime are used^47^. A nanofluidic power generator device of a power density of 705 W/m^2^ using a KCl concentration gradient in a nanochannel of the dimensions 715 nm × 350 nm × 40 nm was demonstrated by Zhang et al.^48^—fabricated in a silicon chip. However, care must be taken when comparing softlithography devices with silicon applications. Handling the PDMS carefully during the bonding process is crucial to not bend or collapse nanostructures on the final substrate. Silicon chips are more reliable and stable during operation, but PDMS is cost-effective and offers the advantage of fast replacement. The lateral resolution of the two-photon effect can be pushed to the limit by using the STED principle.^49^ Using photoresists consisting of tri- and tetra-acrylates and 7 diethylamino-3-thenoylcoumarin and surrounding the focal spot with a depletion beam pushes the polymerized features size to 55 nm at a resolution of 120 nm, which shifts two-photon lithography technology further towards EBL resolution for nanofabrication.

## Methods

### Sample preparation and development

In all, 25 µm of SU-8 were spin coated (BRUKER) at 3000 rpm onto a silicon wafer. The wafer was soft baked and treated according to the protocol of the photoresist distributor (Microchem). A microfluidic mask pattern was then projected onto the photoresist for 50 s with the setup described in Challa et al.^50^. The wafer post baked at 95 °C so that the interfaces between developed and undeveloped regions become visible. After the two-photon writing process the whole wafer was again baked at 95 °C and finally rinsed with PGMEA and IPA.

### Two-photon lithography setup

To produce the high intensities needed for two photon excitation to occur, a Menlo System C-Fiber 780 HP Er:doped fiber oscillator with a repetition rate of 100 MHz with integrated amplifier and second harmonic generation was used as light source of the two photon system. The laser pulse width is 120 fs. The setup is optimized to produce nanoscale features on a 100 µm × 100 µm field. The beam is expanded to fill the whole back aperture of the objective lens (Leica, PL APO, Magn. 100×, NA = 1.4, oil) using a Thorlabs beam expander (AR coated: 650–1050 nm, GBE05-B). To make positioning of the focal spot over a whole 3″ wafer possible, two PI linear-precision stages (M-404.2PD, Ball screw, 80 mm wide, ActiveDrive) were mounted perpendicular on top of each other with a suitable adaptor plate, connected to two individual PI Mercury DC Motor Controllers (C-863.11, 1 Channel, wide range power supply). On top, a PI NanoCube with a travel range of 100 µm × 100 µm × 100 µm is mounted for high precision movement (pos. resolution: 1 nm) of the sample during the writing process. We focus on the wafer/photoresist interface using the back-reflection of the laser beam at low intensity (where polymerization will not occur), and capture the image via a USB-camera (µEye ML, Industry camera, USB 3.0) with a tube lens (Thorlabs AC 254-100-A-ML, BBAR coating 400–700 nm, *f* = 100.0 mm) mounted above the objective. The objective is mounted onto a Thorlabs Z812B stage to allow wider travel range in *z* when moving over the wafer. The laser modulation is controlled by an acousto-optic modulator by AA Optoelectronics mounted after the laser output port connected to a fast switching power supply (ISOTECH,DC power supply, IPS 33030). In order to provide an open-source setup all the control programming is realized in Python as well as the automated writing of a calibration assay of the system.

## Conclusion

We integrated successfully nanofluidic functionalities into microfluidic devices by combining two-photon lithography with mask based UV lithography on a silicon wafer. Pre-exposed areas generated first through conventional UV lithography undergo a refractive index change which can then be used to align the two-photon writing process. We showed that the two-photon lithography setup presented here is capable of producing features down to 230 nm lateral width on a silicon wafer surface in SU-8 photoresist and successfully integrated 420 nm wide nanochannels into a microfluidic master design. By ascending progressively the exposure voxel, reliable nanochannel molds of sub 100 nm in height were fabricated. This regime has the potential to produce PDMS devices that are comparable to EBL or RIE-etched chips. In contrast to other techniques e.g nanochannel fabrication by cracking, where mechanics and reagents define the shape of the formed nano junctions – two-photon lithography allows the integration of arbitrary nano-sized patterns and complex shapes including varying channel sizes into microfluidic devices. We verified the reliability of the fabrication process by comparing SEM images of a SU-8 calibration sample with TIRF fluorescence super-resolution imaging in the final PDMS devices. Further improvements in resolution of the process could be achieved by a change in photoresist composition or post-processing of the photoresist via temperature and plasma treatment to thin out written structures. We hope to provide with this method a fast, reliable and flexible pathway for nanofluidic device fabrication to enable readily the addition of nanofluidic features to conventional devices.

## Supplementary information

single_molecule_Rhod6G_video

Supplementary Information
