# Supplementary material for: Scalable integration of nano-, and microfluidics with hybrid two-photon lithography
Source: Microsyst Nanoeng. 2019 Sep 9;5:40. doi: 10.1038/s41378-019-0080-3 (PMC6799807; doi:10.1038/s41378-019-0080-3)
Supplement: Supplementary file 2 — Supplementary Information [file 41378_2019_80_MOESM2_ESM.pdf]

Supplementary Information

Supplementary figure 1

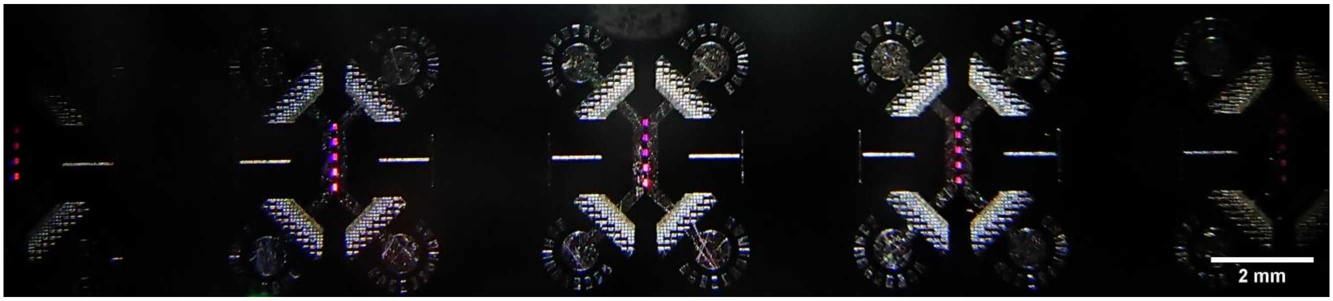

Figure S1. Bright-field microscopy image showing wafer-scale integration of nanofluidic channels into a microfluidic master using hybrid two-photon lithography in SU-8. Microfluidic reservoirs of independent devices were joined with nanochannels and demonstrate the scalability of the technique using the optical setup controlled by the available open-source software in Python. (Supplementary figure 2) The nanochannels can be easily observed due to their grating-like diffraction pattern when illuminated with a white light source.

Supplementary figure 2

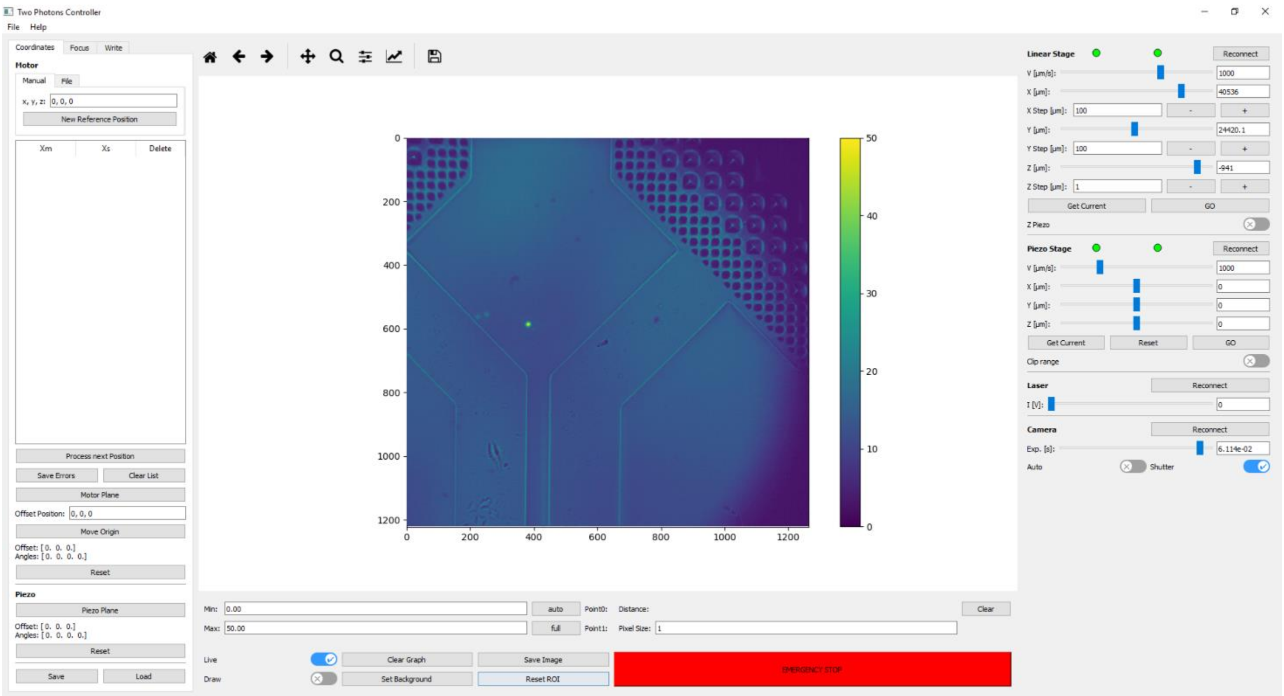

Figure S2. GUI of the Python control software developed for this system. The software enables wafer-scale automated writing of 2-photon structures and provides handling of autofocussing, laser writing, stage movement, piezo movement, sample tilt correction and camera acquisition. The control software is provided as open-source package here: https://doi.org/10.5281/zenodo.3229115 and can be modified or used by other researchers to build their own open-source 2-photon system based on the key hardware components described in the methods section.

Supplementary figure 3

Decreasing the power of the laser beam at constant offset allows the control of channel widths arbitrarily from micron to the nano range (see Fig.S3 A). According to the filling factor of the intended application or in this case intended channel size - the necessary power parameter can be read out from the assay. Fig.S3 B shows a line profile plot of low NA 2P-written lines fabricated at 400 µm/s and illustrate how by variation of the laser power from 58.5 mW to 70.0 mW, the channel width can be controlled from micron to 230 nm in size at the same offset of e.g. at -2.51 µm.

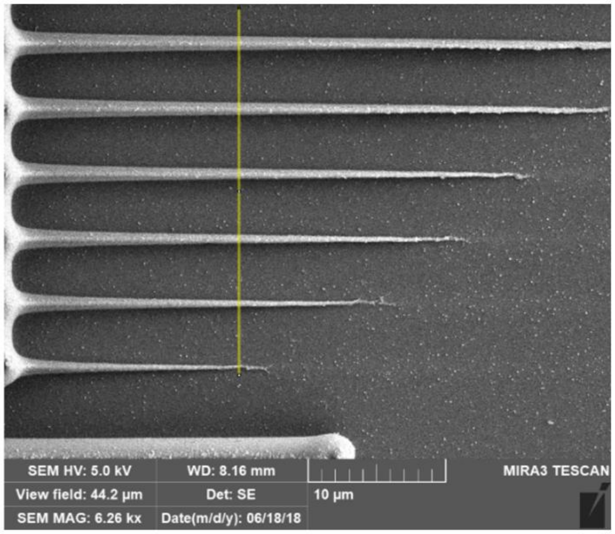

A

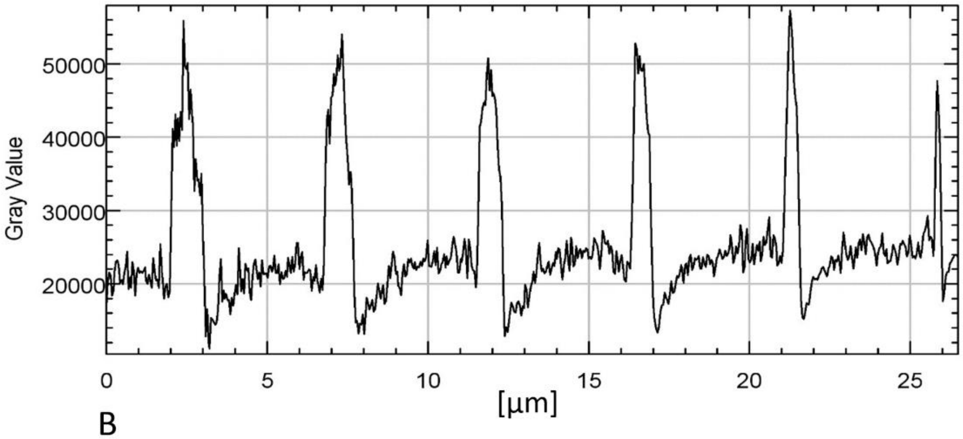

B

Figure S3. (A) SEM image of NA-matched calibration assay written at 400 µm/s with laser intensities ranging from 58.5 mW to 70 mW and offset from -2 µm to -5 µm; (B) Line profile along power axis of calibration assay as indicated in (A). At constant offset and scanning speed, the channel width can be controlled in the sub-micron regime down to 230 nm.

Supplementary figure 4

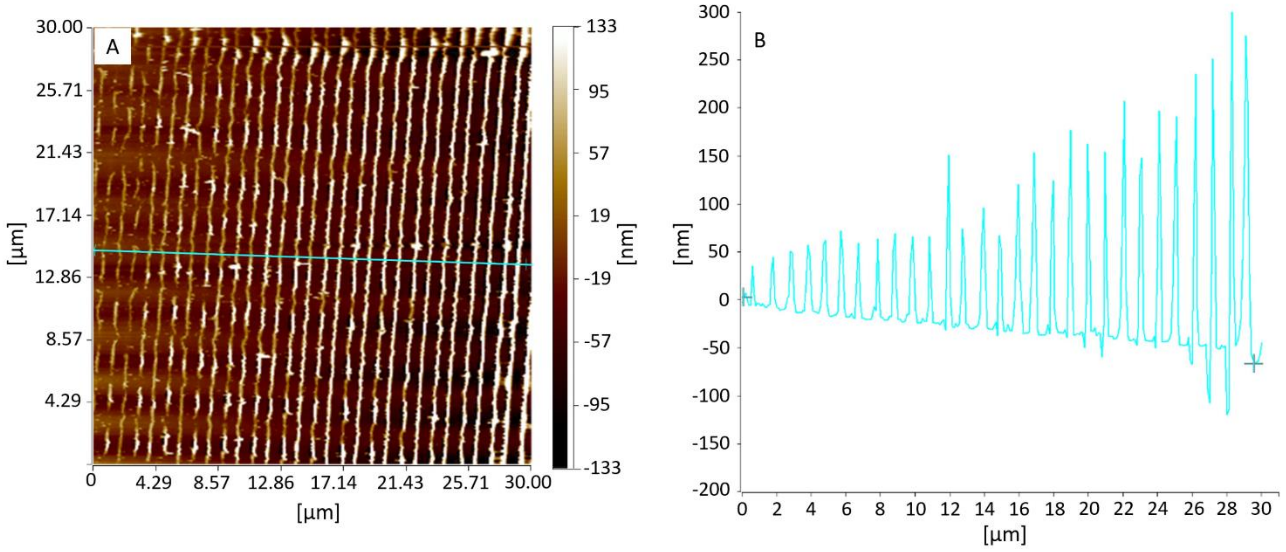

Figure S4. The translational motor stages were used to write lines into SU-8 at 120 mW and 400 µm/s - slowly deepening the focus into the wafer, following a meander pattern with a spacing of 1 micron. AFM data shows polymerized lines with a height down to 35 nm, but also show detachment due to the linear stages not moving smooth enough for this fabrication regime.

Supplementary figure 5

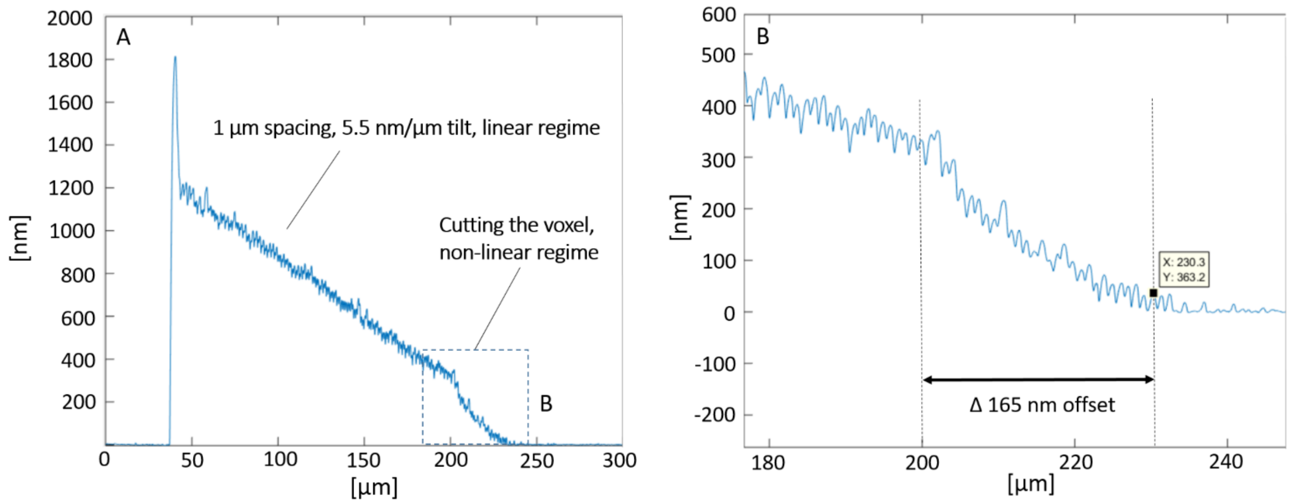

Figure S5. (A) Height profile plot acquired with a Dektak BRUKER profilometer. A sample wafer with at 25 µm thick SU-8 layer was mounted in the system. The laser was focused onto the surface and the motor stages used to write a meander pattern with 1 µm spacing, under a slope of 5.5 nm/µm, at a writing speed of 400 µm/s and 120 mW laser power into the wafer. As can be seen, the printed heights range from micron to the nanoscale and show a linear slope. Especially, when reaching the sub 300 nm regime a non-linear dependence on the offset can be found when the polymerization inducing voxel is truncated by the silicon surface; (B) Detailed view of the non-linear regime shows regions with heights down to 36 nm. This measurement implies the need for highly precise positioning for reliable fabrication on the nanoscale. The piezo technology used in this setup has a positioning resolution of 1 nm in closed loop mode and allows therefore precise control of channel heights within this fabrication window.

Supplementary figure 6

While 2P-writing of features above 300 nm provides proper moulds for soft lithography, we observed the formation of multiple lines at positions where the voxel was deepened into the wafer. We assumed that by overfilling the back aperture of the high-NA objective an interference pattern is formed by the high spatial frequency components of the focused laser beam. These reflections cause major difficulties when nanofluidic channels below 300 nm height need to be written onto silicon wafers. To detect the interference patterns we measured the 3D PSF of the system by overfilling the back aperture of the objective with a 5:1 beam expander and measured the far field back reflection of the laser beam from the silicon wafer surface. We acquired an image stack with 50 nm step size while lowering the laser beam at low intensity into the wafer. From an orthogonal slice of the acquired z-stack (see Fig. S6 A), one can see the interference patterns above the focal spot which are caused by high spatial frequency components undergoing reflection due to the refractive index change from SU-8 to silicon. A pinhole in the laser beam path was adjusted so that the reflected interference patterns were minimized and another image stack was acquired as shown in Fig. S6 B. By comparing the z-profile plots (see Fig. S6 C,D) of both PSFs it becomes obvious that the intensity maxima above the wafer surface are reduced. Therefore, we incorporated a variable beam expander (Thorlabs, BE02-05-B) into the system and repeated the calibration assay and adjusted the parameters accordingly so that voxel truncation occurs. We found that by reducing the numerical aperture we can preserve similar high resolution 2P-printing capabilities and reduce back-reflections, which enables reliable channel fabrication at arbitrary heights from micron to the sub 300 nm regime on a silicon wafer. The improvement is demonstrated, by changing the filling factor of a high NA objective and comparing calibration assays using SEM imaging. As one can see in Fig. S6 E multiple foci cause written lines to rip apart and renders them unsuitable for soft lithography. The improved and smooth printing capability can be seen in the SEM image (Fig. S6 F), where lines decrease in width and height consistently without detaching parts.

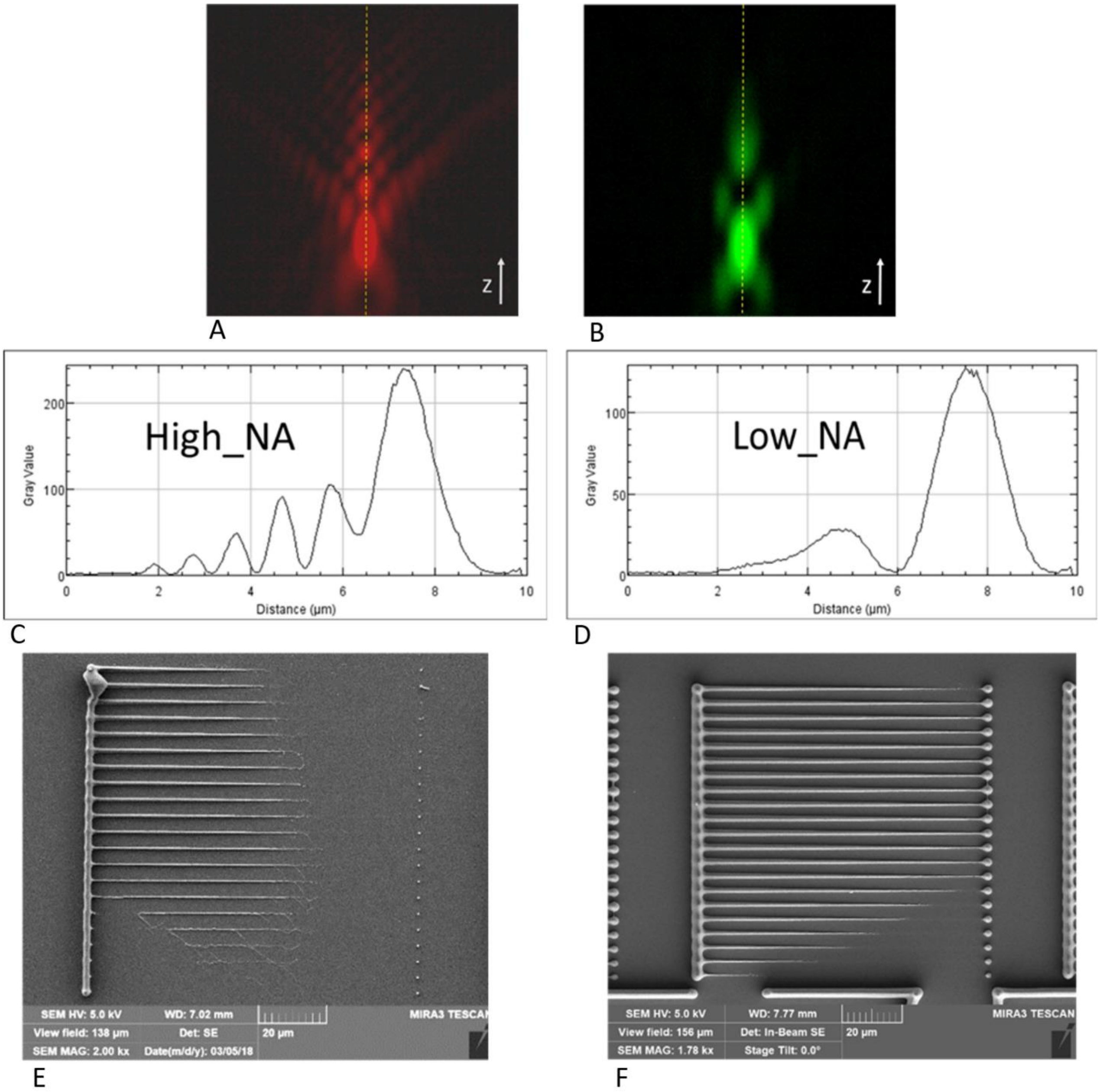

Figure S6. (A) the orthogonal projection of the reflected PSF shows the interference patterns above the wafer surface; (B) Reducing the beam diameter coupled into the objective reduces the interference generated along the optical axis; (C) Line profile along z-axis for high NA case; (D) Line profile along z-axis for adopted beam diameter; (E) Calibration assay written at 200 µm/s, with an offset range from 0 µm to -3 µm using an overfilled back aperture; (F) Calibration assay written at 200 µm/s with an offset from -2 µm to -5 µm into the silicon wafer and adjusted beam diameter before the objective.
